# Supplementary figures and images for: Autonomous metabolic reprogramming and oxidative stress characterize endothelial dysfunction in acute myocardial infarction
Source: eLife. 2023 Nov 28;12:e86260. doi: 10.7554/eLife.86260 (PMC10871716; doi:10.7554/eLife.86260)

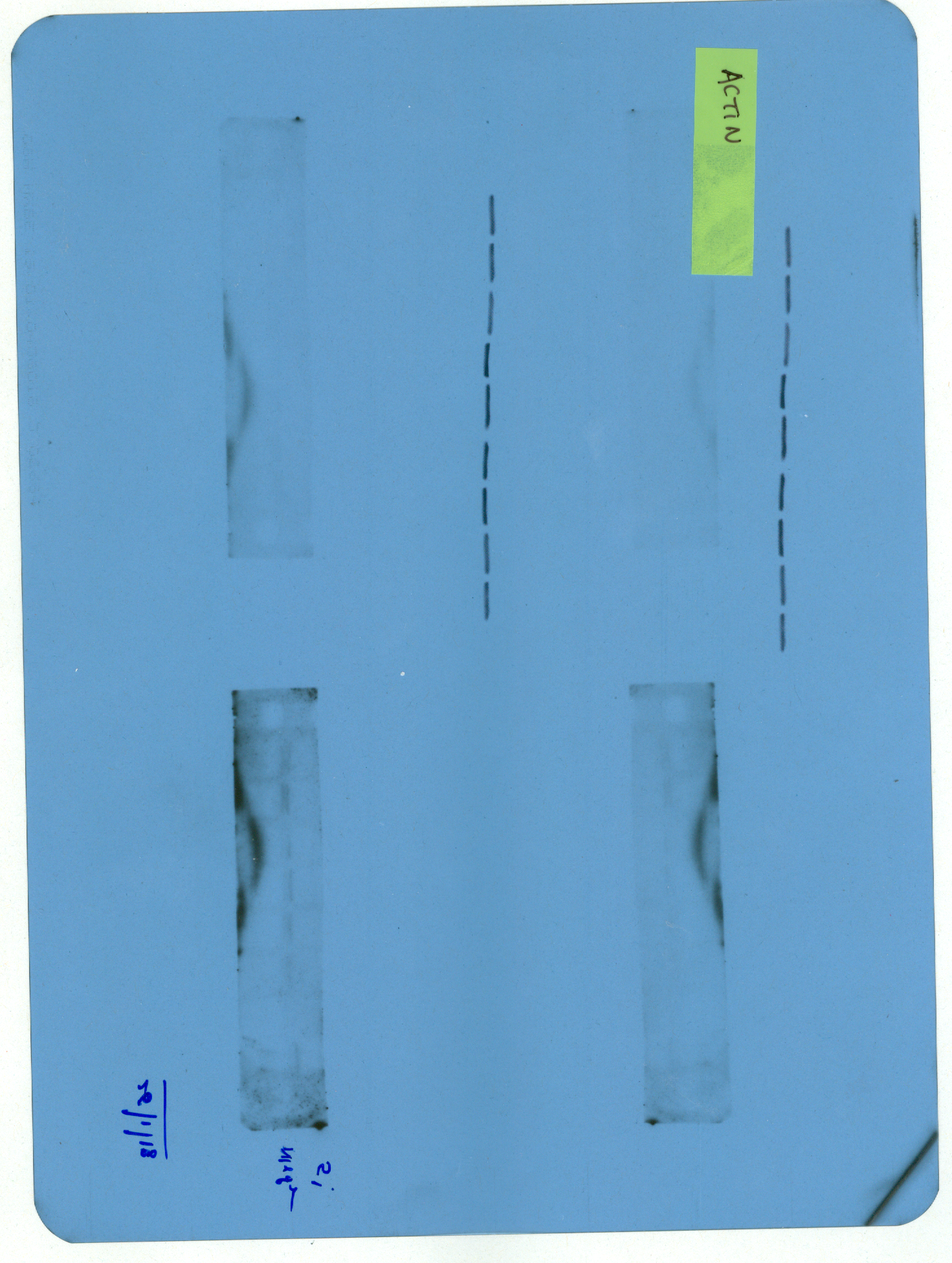

Supplement: Figure 1—source data 1. [file elife-86260-fig1-data1.zip › Fig 1B/b-actin/actin 0001.tif]

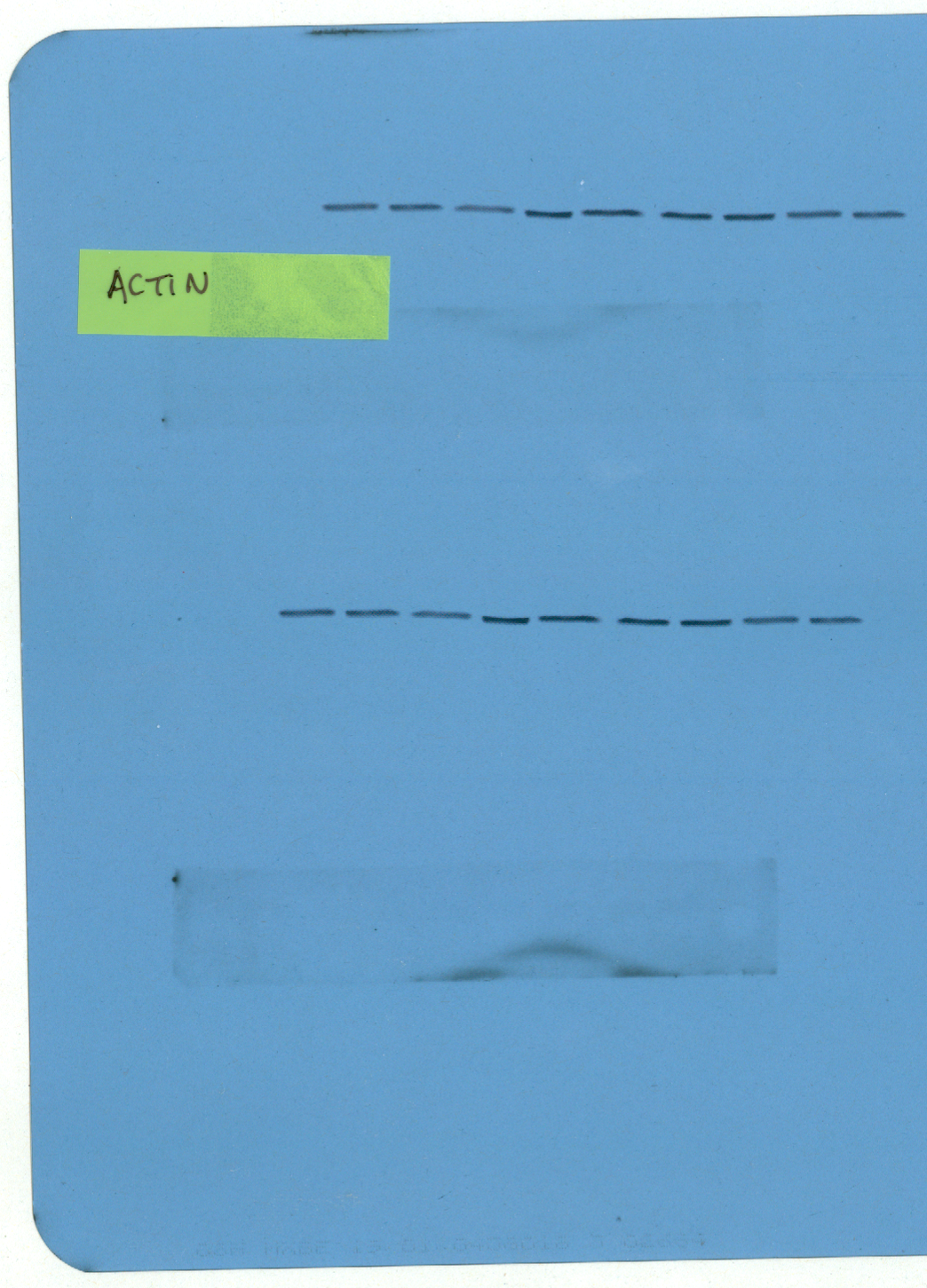

Supplement: Figure 1—source data 1. [file elife-86260-fig1-data1.zip › Fig 1B/b-actin/actin cropped0001.tif]

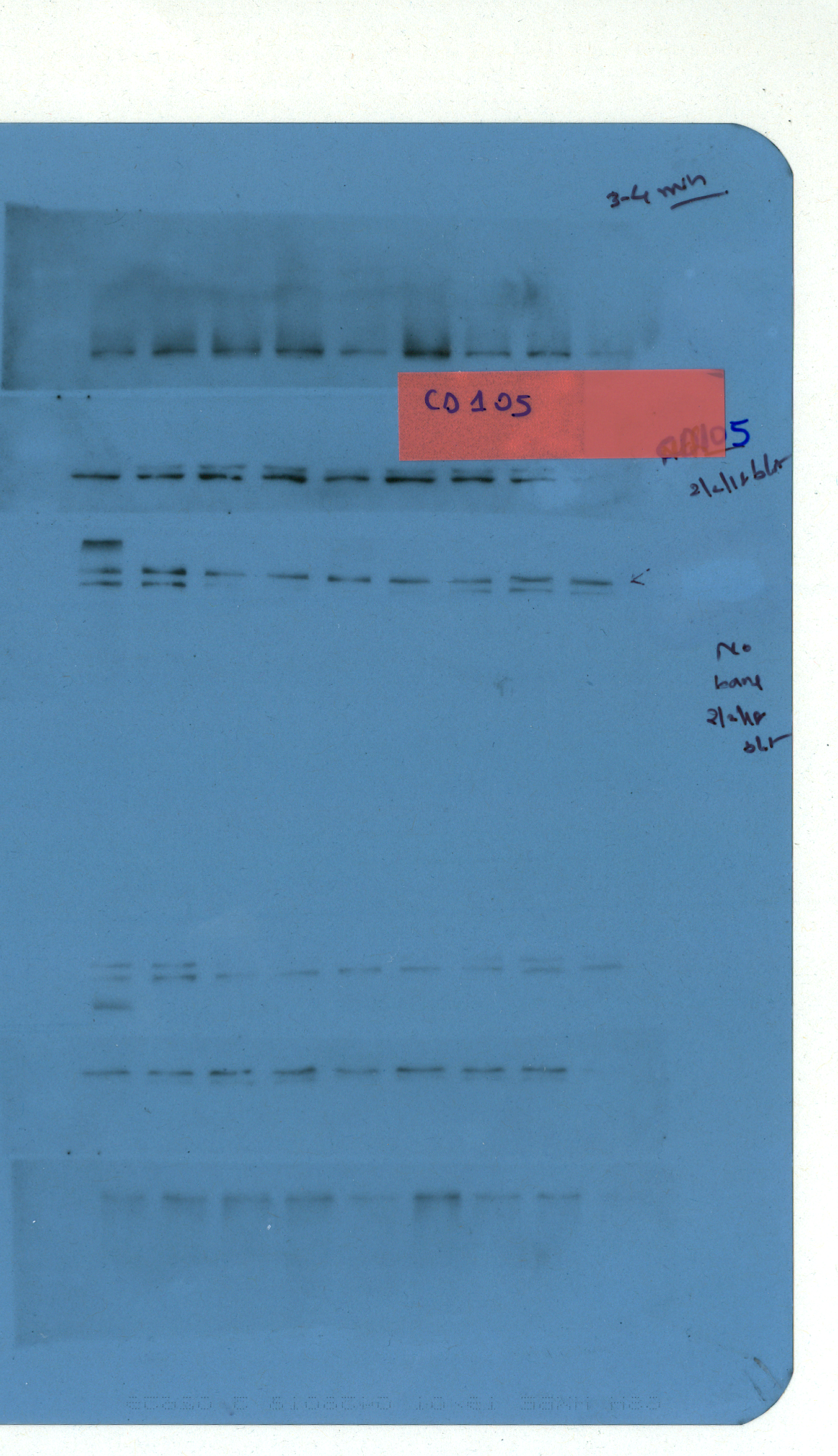

Supplement: Figure 1—source data 1. [file elife-86260-fig1-data1.zip › Fig 1B/CD 105/cd105 cropped0001.tif]

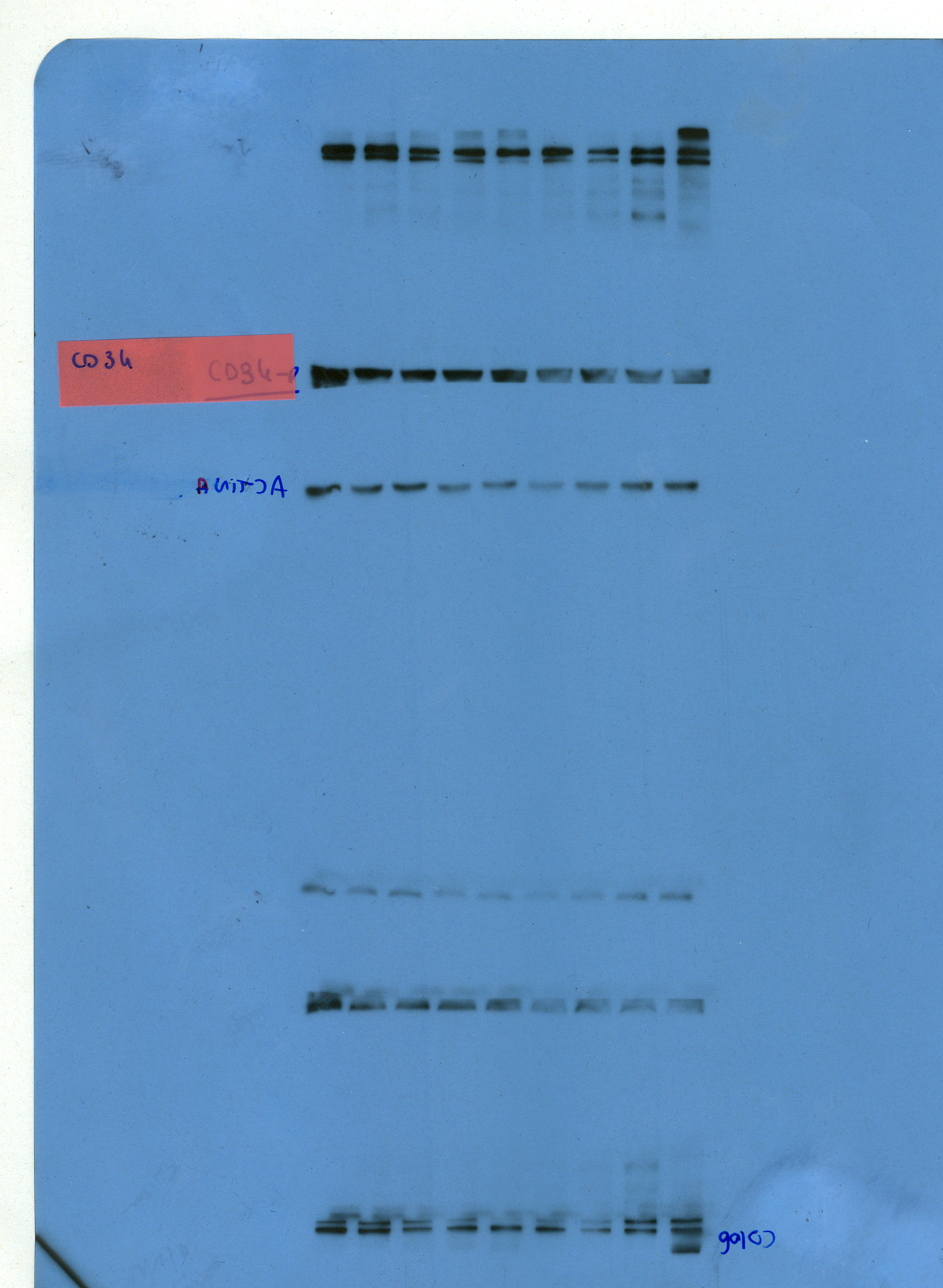

Supplement: Figure 1—source data 1. [file elife-86260-fig1-data1.zip › Fig 1B/CD 34/CD34 0001.tif]

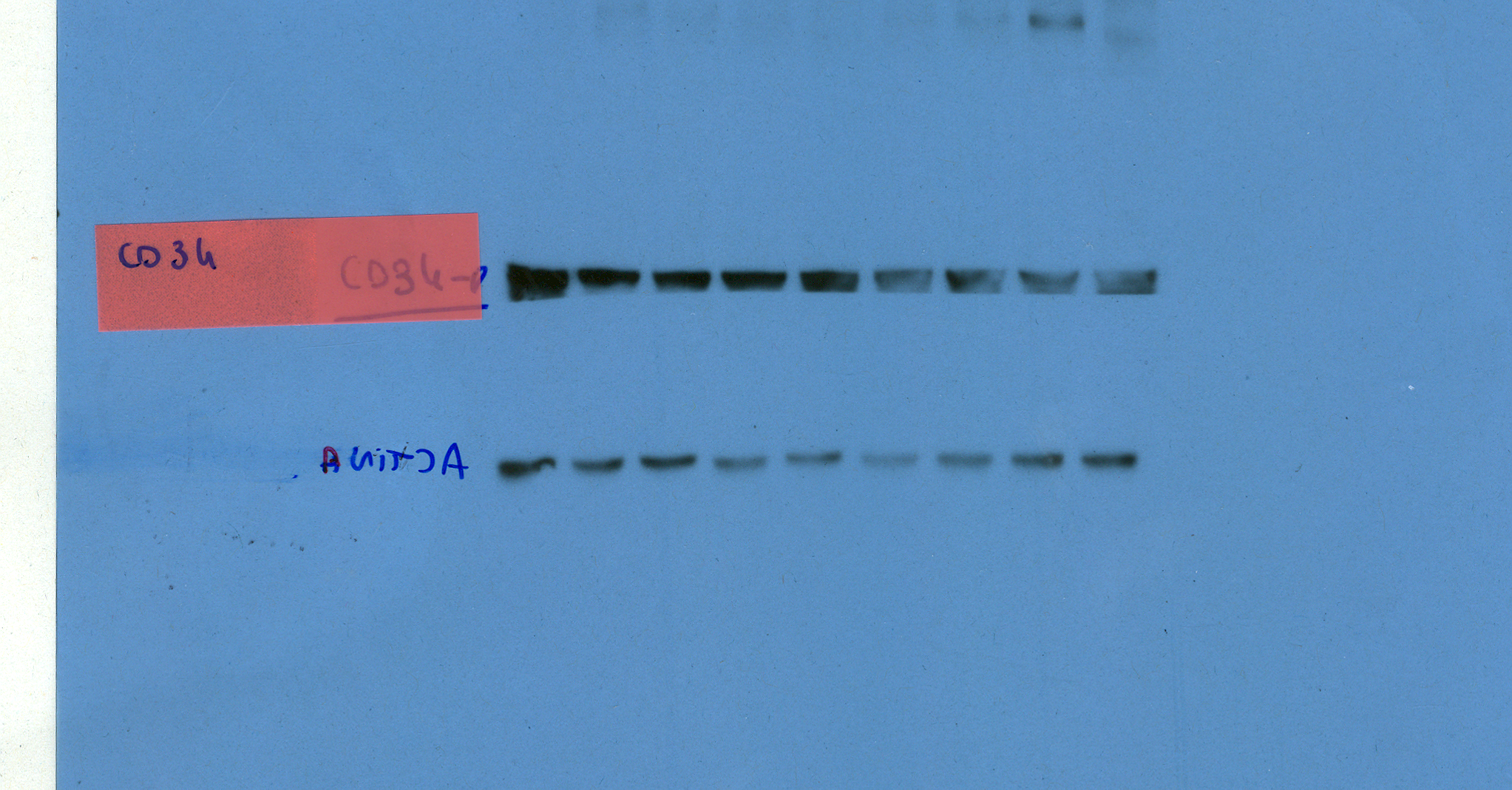

Supplement: Figure 1—source data 1. [file elife-86260-fig1-data1.zip › Fig 1B/CD 34/CD34 cropped0001.tif]

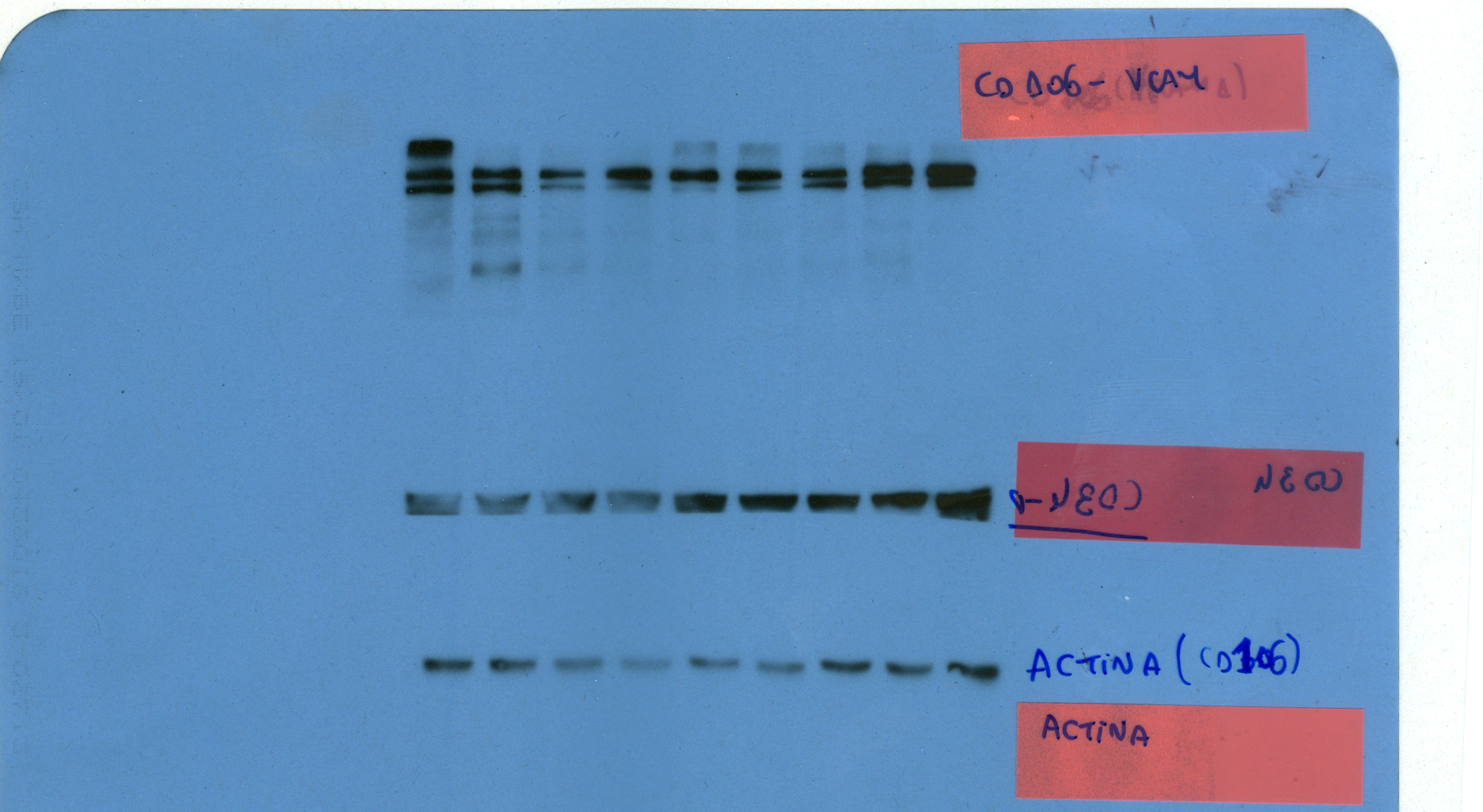

Supplement: Figure 1—source data 1. [file elife-86260-fig1-data1.zip › Fig 1B/CD106-vecam/cd106(VECAM) and ACTIN crop0001.tif]

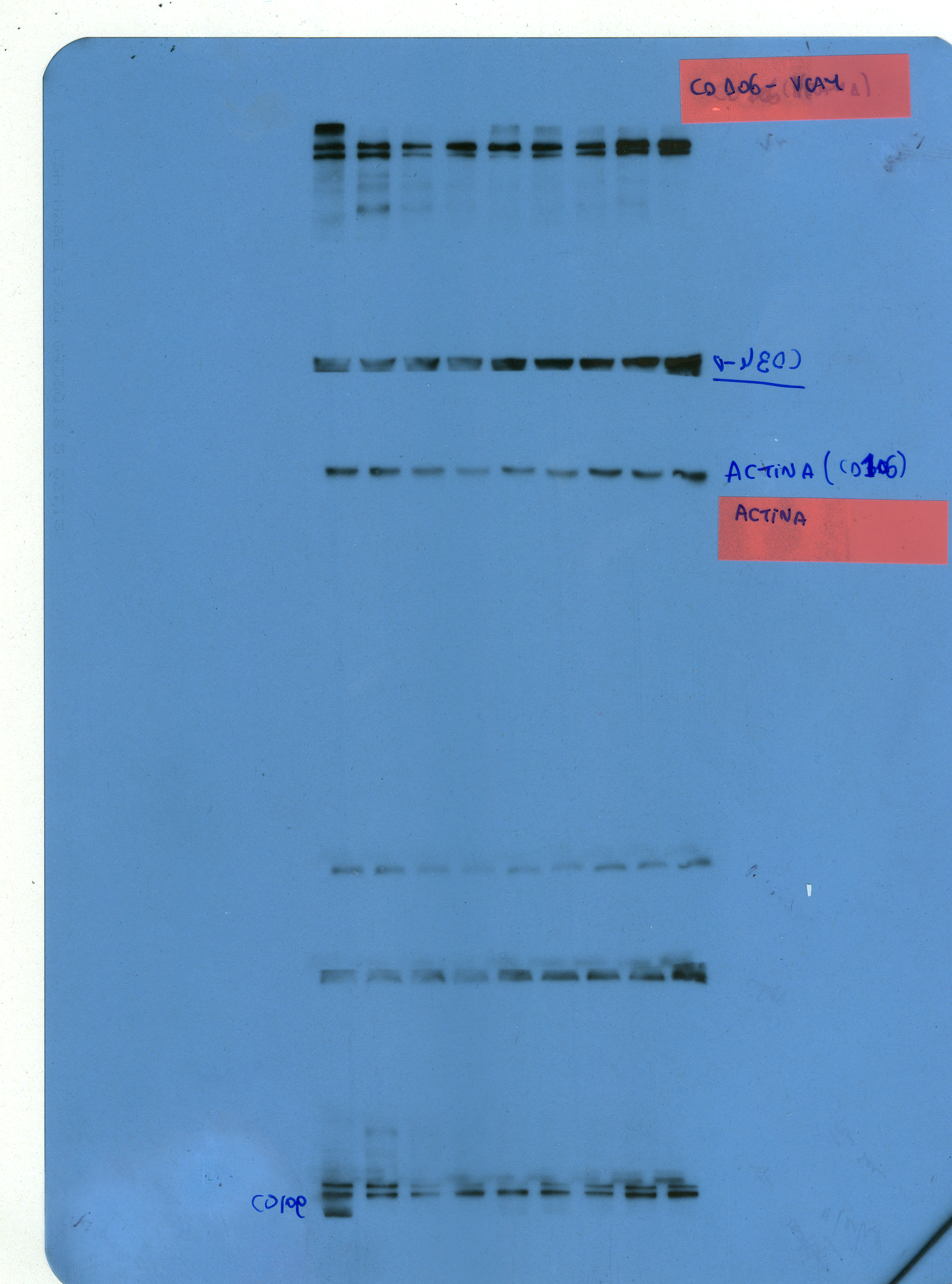

Supplement: Figure 1—source data 1. [file elife-86260-fig1-data1.zip › Fig 1B/CD106-vecam/cd1060001.tif]

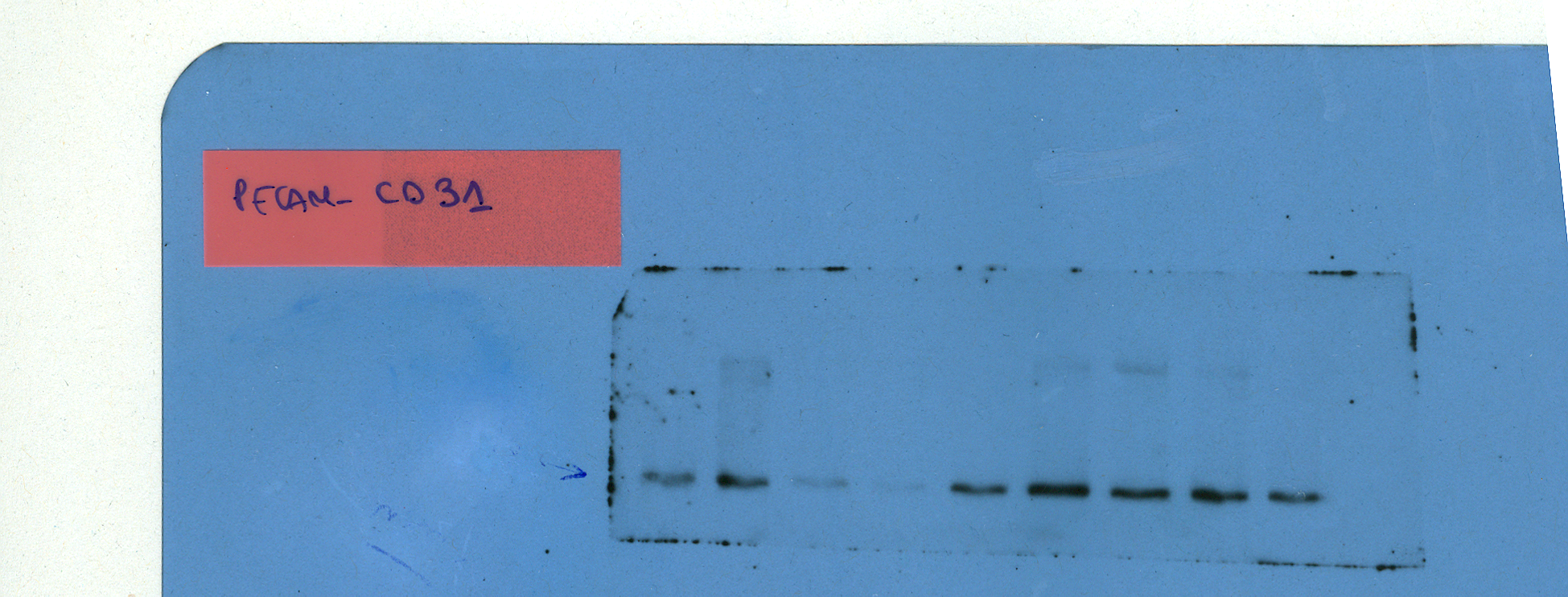

Supplement: Figure 1—source data 1. [file elife-86260-fig1-data1.zip › Fig 1B/CD31-pecam/cd31 pecam crop 20001.tif]

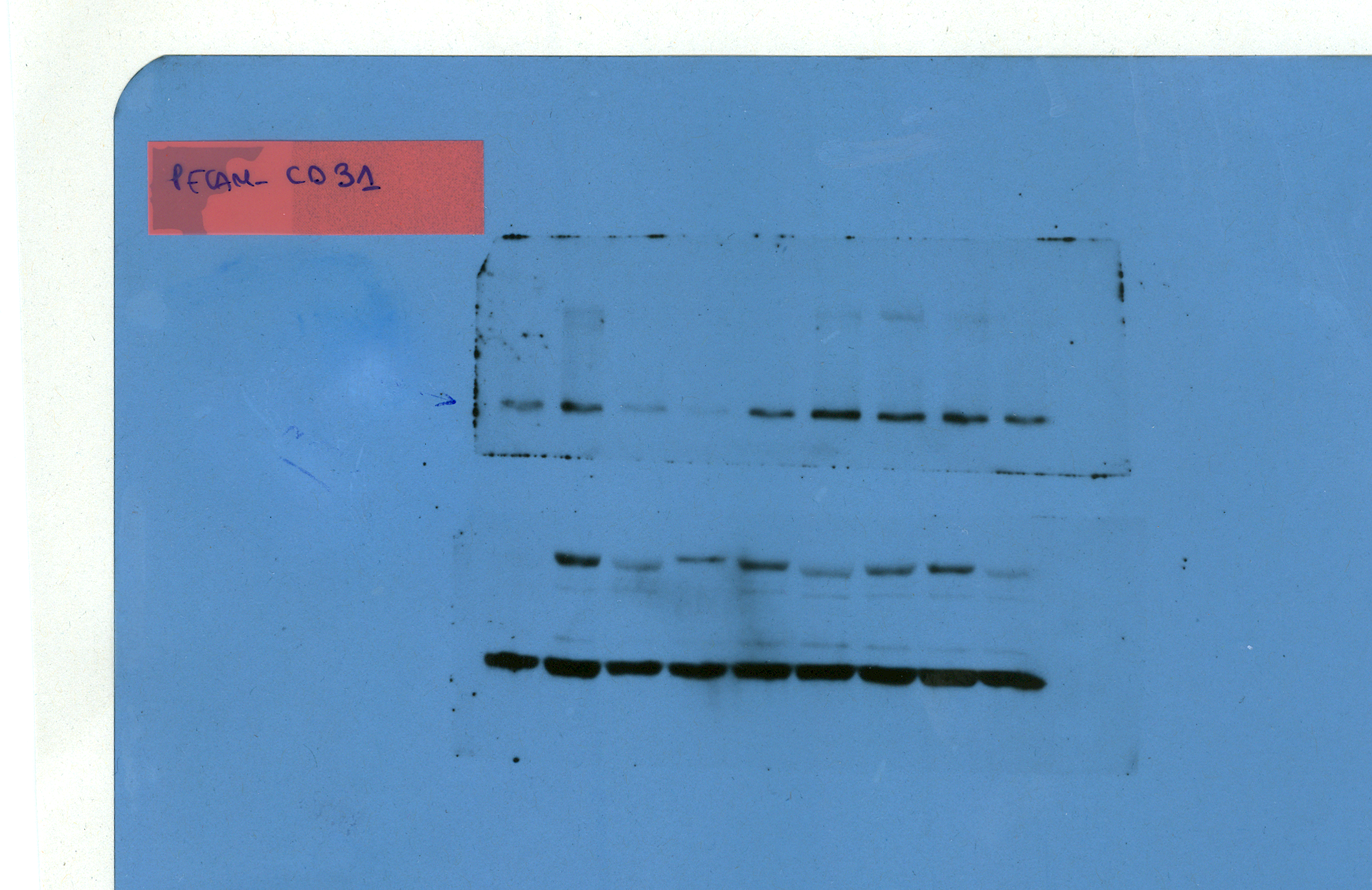

Supplement: Figure 1—source data 1. [file elife-86260-fig1-data1.zip › Fig 1B/CD31-pecam/cd31 pecam cropped0001.tif]

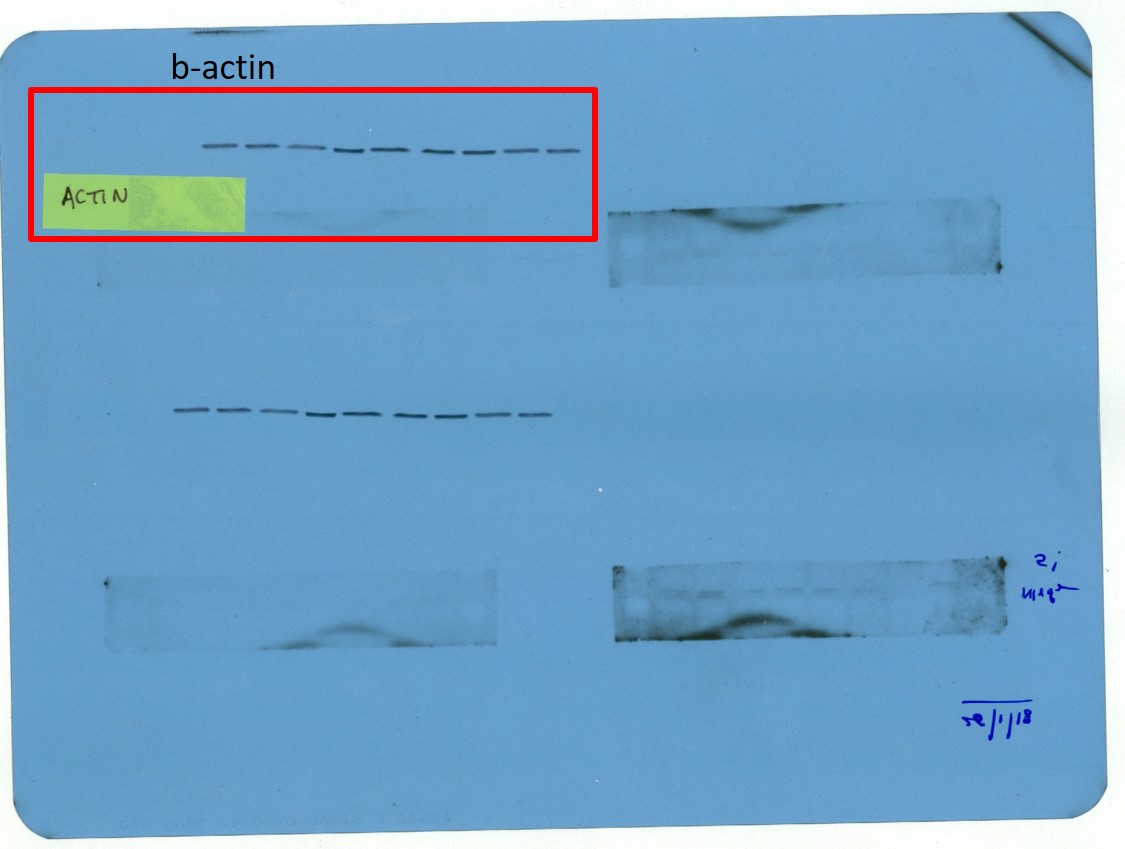

Supplement: Figure 1—source data 2. [file elife-86260-fig1-data2.zip › Fig1 B_source data NEW/b-actin/b-actin jpeg.jpg]

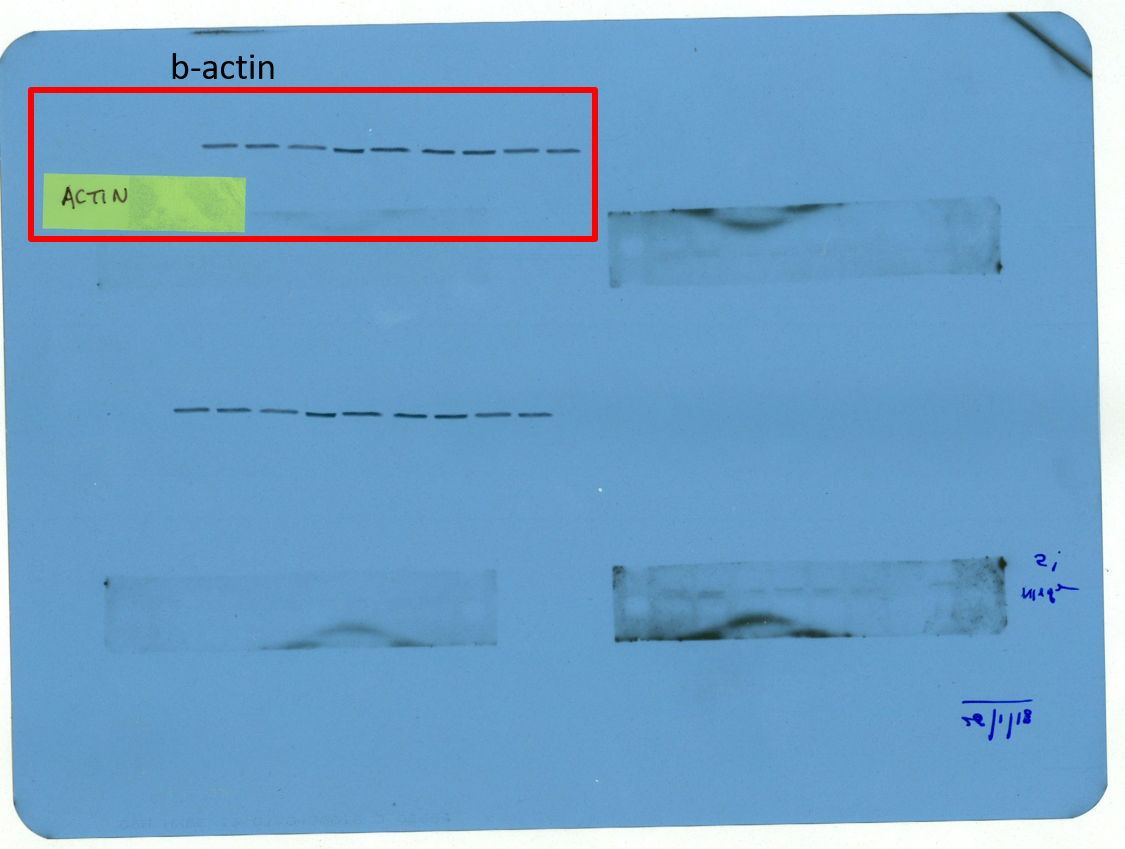

Supplement: Figure 1—source data 2. [file elife-86260-fig1-data2.zip › Fig1 B_source data NEW/b-actin/b-actin.tif]

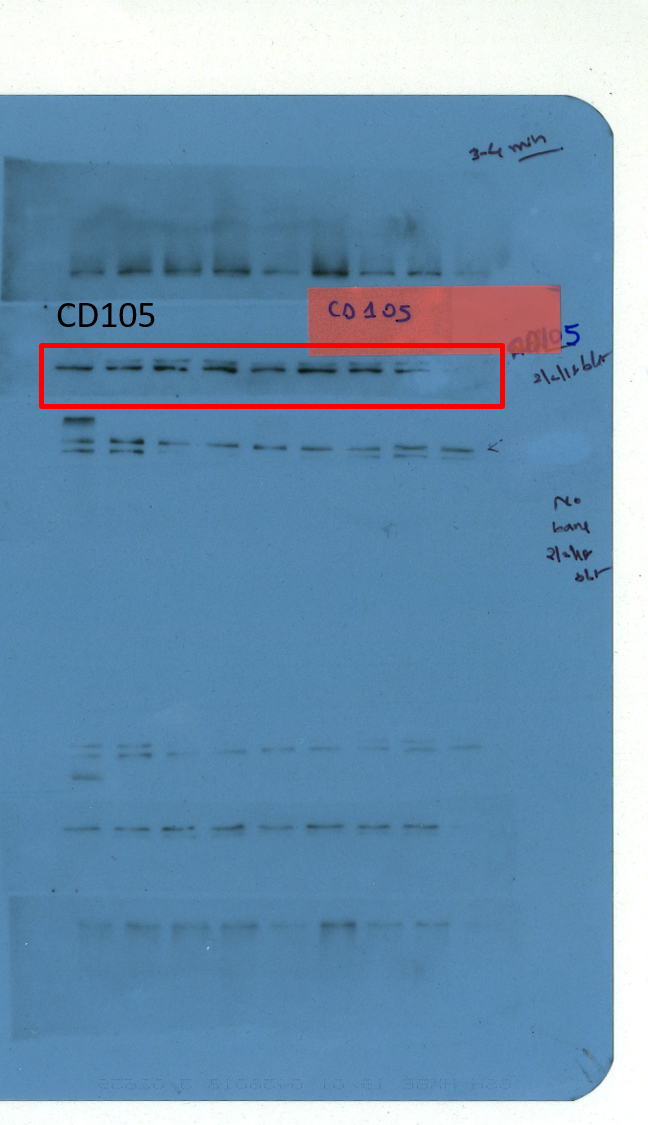

Supplement: Figure 1—source data 2. [file elife-86260-fig1-data2.zip › Fig1 B_source data NEW/CD 105/CD105 jpeg.png]

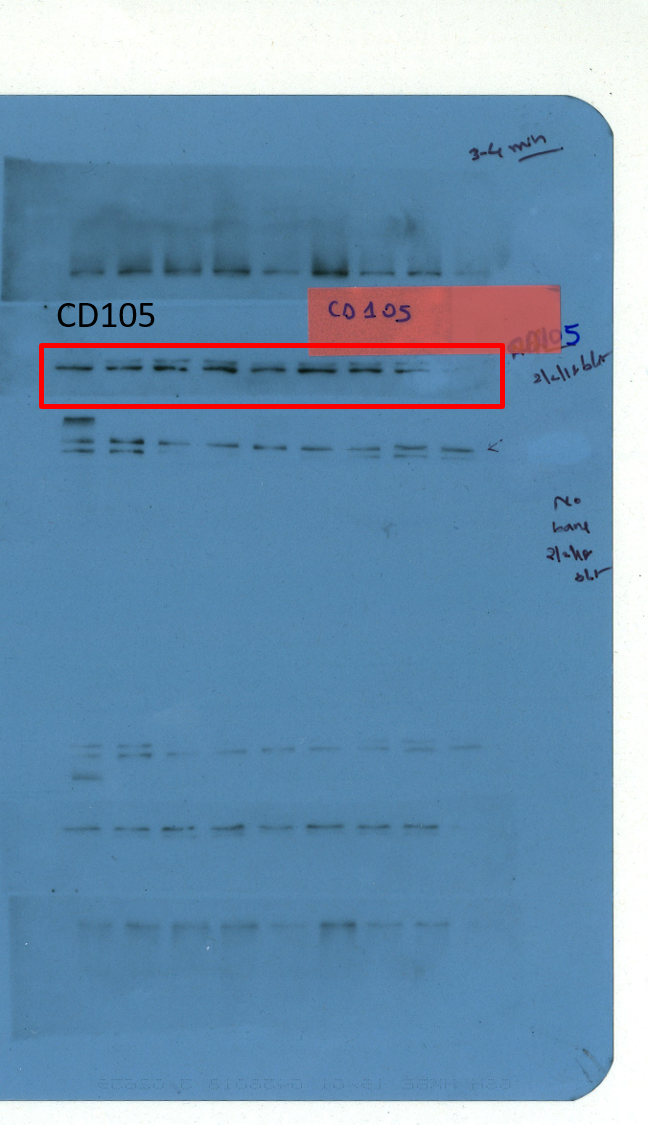

Supplement: Figure 1—source data 2. [file elife-86260-fig1-data2.zip › Fig1 B_source data NEW/CD 105/CD105.tif]

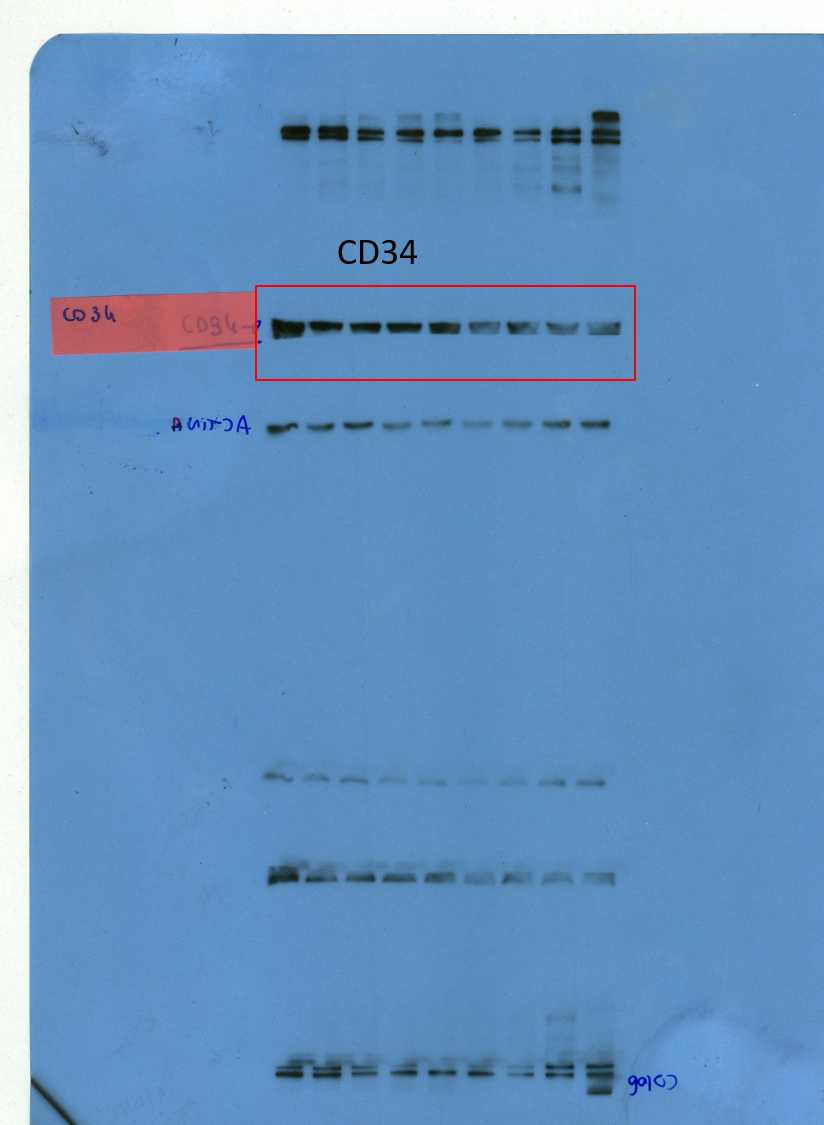

Supplement: Figure 1—source data 2. [file elife-86260-fig1-data2.zip › Fig1 B_source data NEW/CD 34/CD34 .tif]

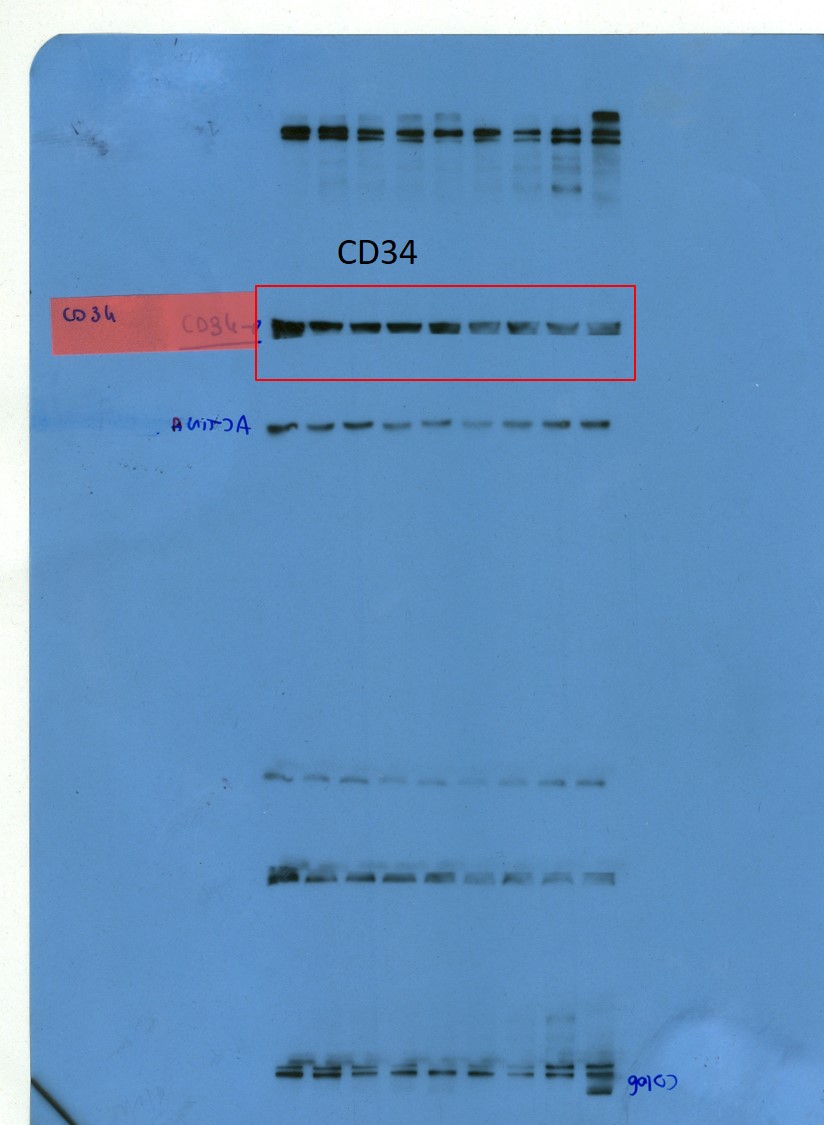

Supplement: Figure 1—source data 2. [file elife-86260-fig1-data2.zip › Fig1 B_source data NEW/CD 34/cd34 jpeg.jpg]

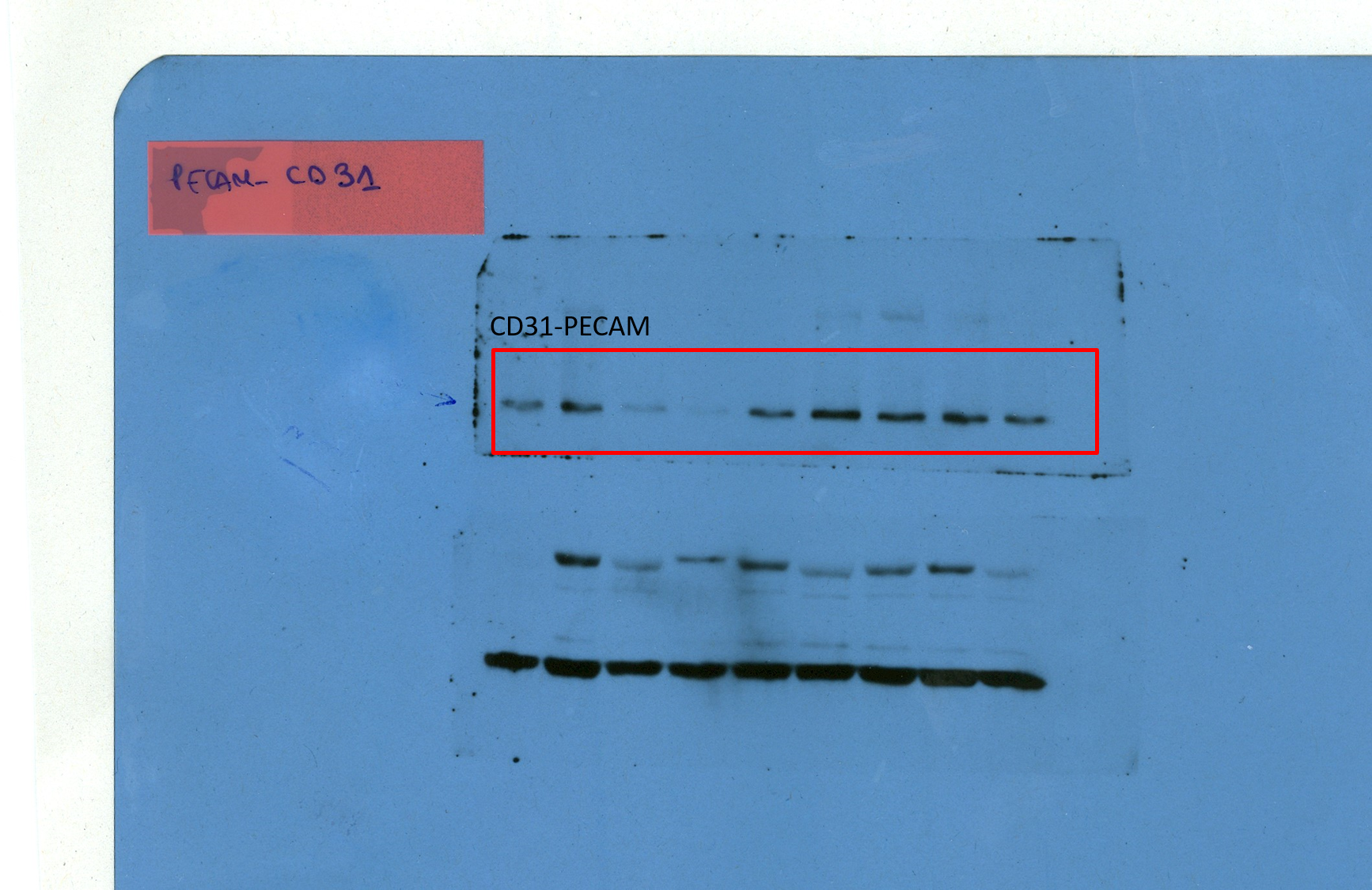

Supplement: Figure 1—source data 2. [file elife-86260-fig1-data2.zip › Fig1 B_source data NEW/CD31-PECAM/CD31-PECAM.tif]

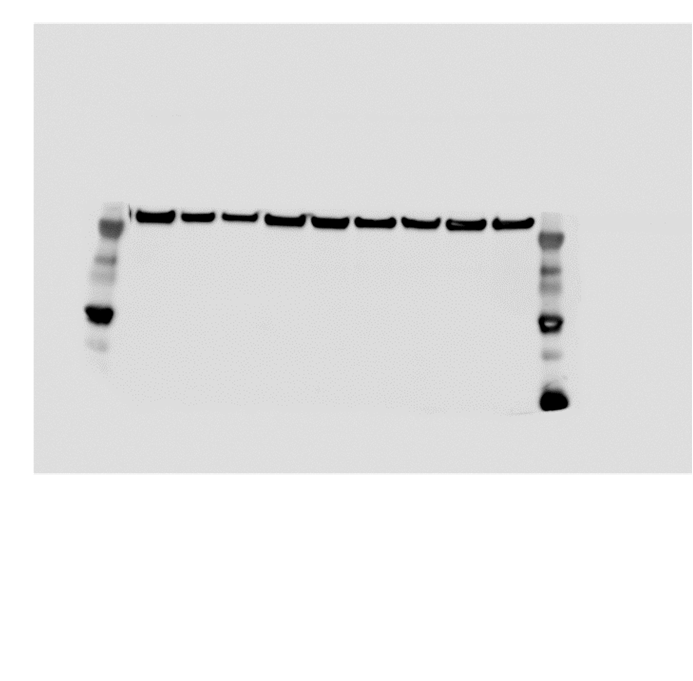

Supplement: Figure 4—source data 1. [file elife-86260-fig4-data1.zip › Fig 4A/Fig4A_pfkfb3 BLOT/b actin pfkfb3.tif]

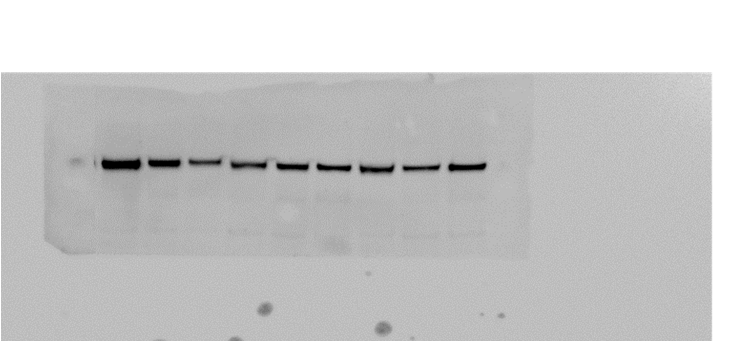

Supplement: Figure 4—source data 1. [file elife-86260-fig4-data1.zip › Fig 4A/Fig4A_pfkfb3 BLOT/pfkfb3_membrane.tif]

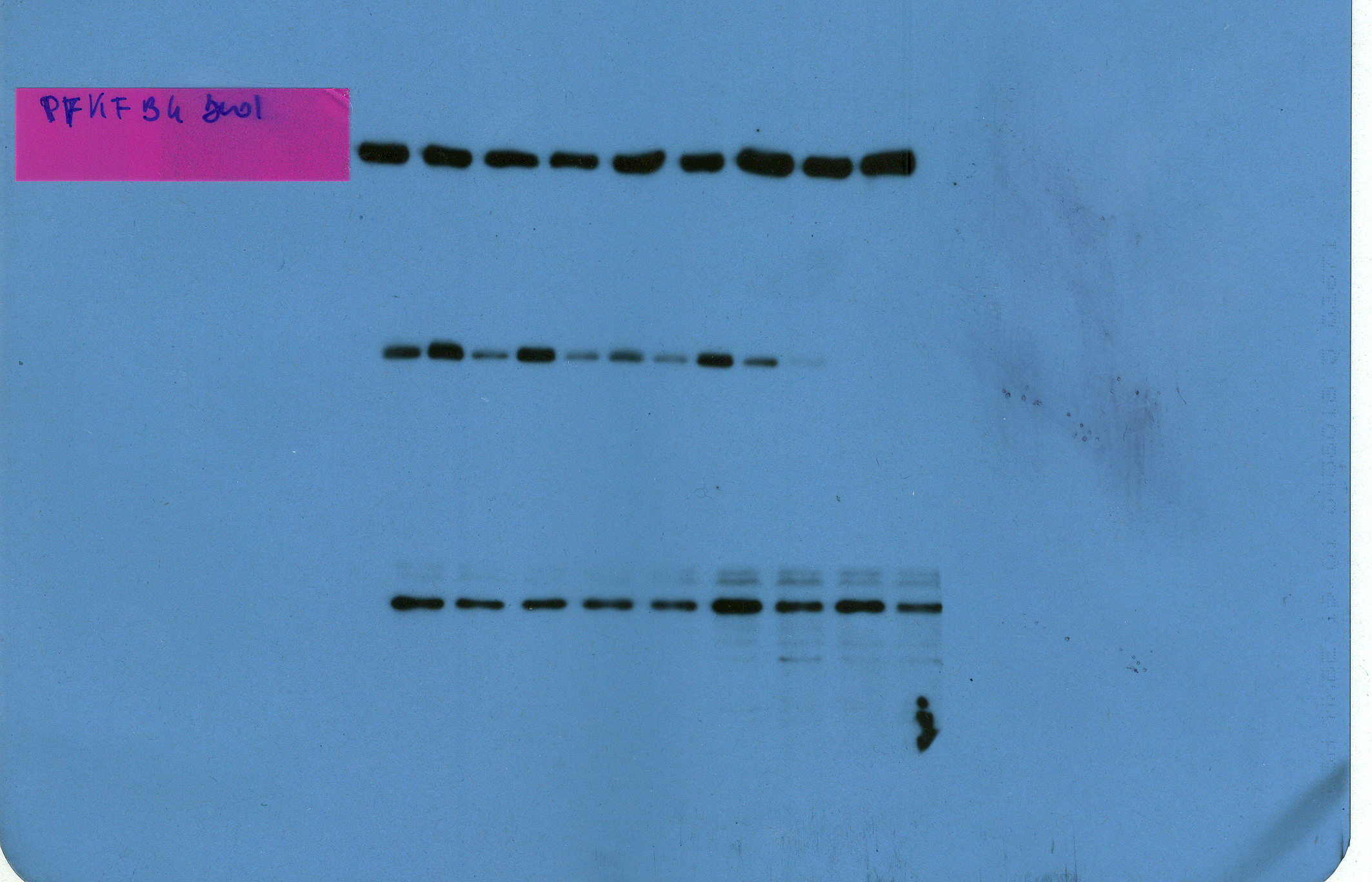

Supplement: Figure 4—source data 1. [file elife-86260-fig4-data1.zip › Fig 4A/Fig4A_pfkfb4 FILM/aPFKFB4 cropped0001.tif]

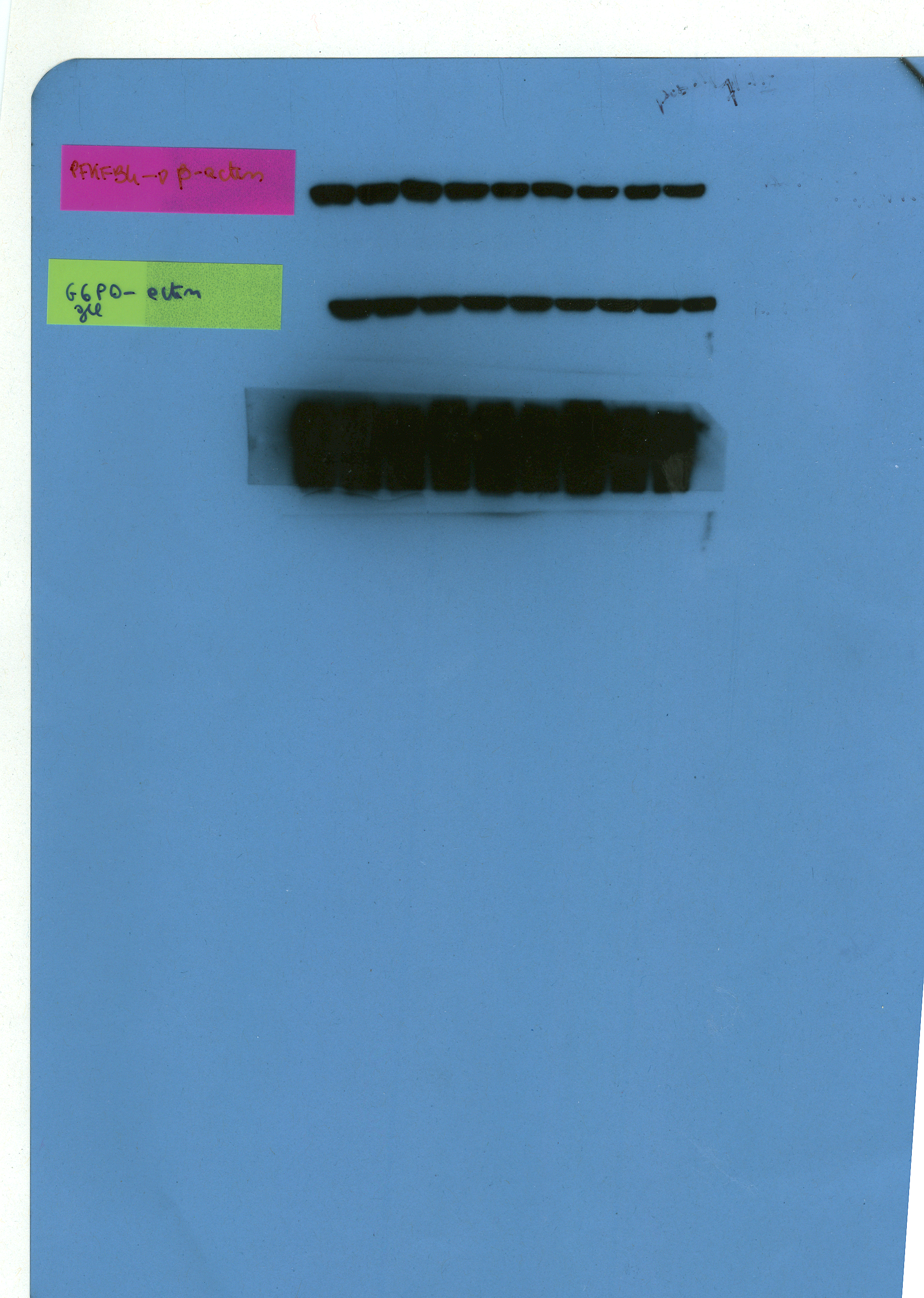

Supplement: Figure 4—source data 1. [file elife-86260-fig4-data1.zip › Fig 4A/Fig4A_pfkfb4 FILM/b-ACTIN pfkfb4 g6pd0001.tif]

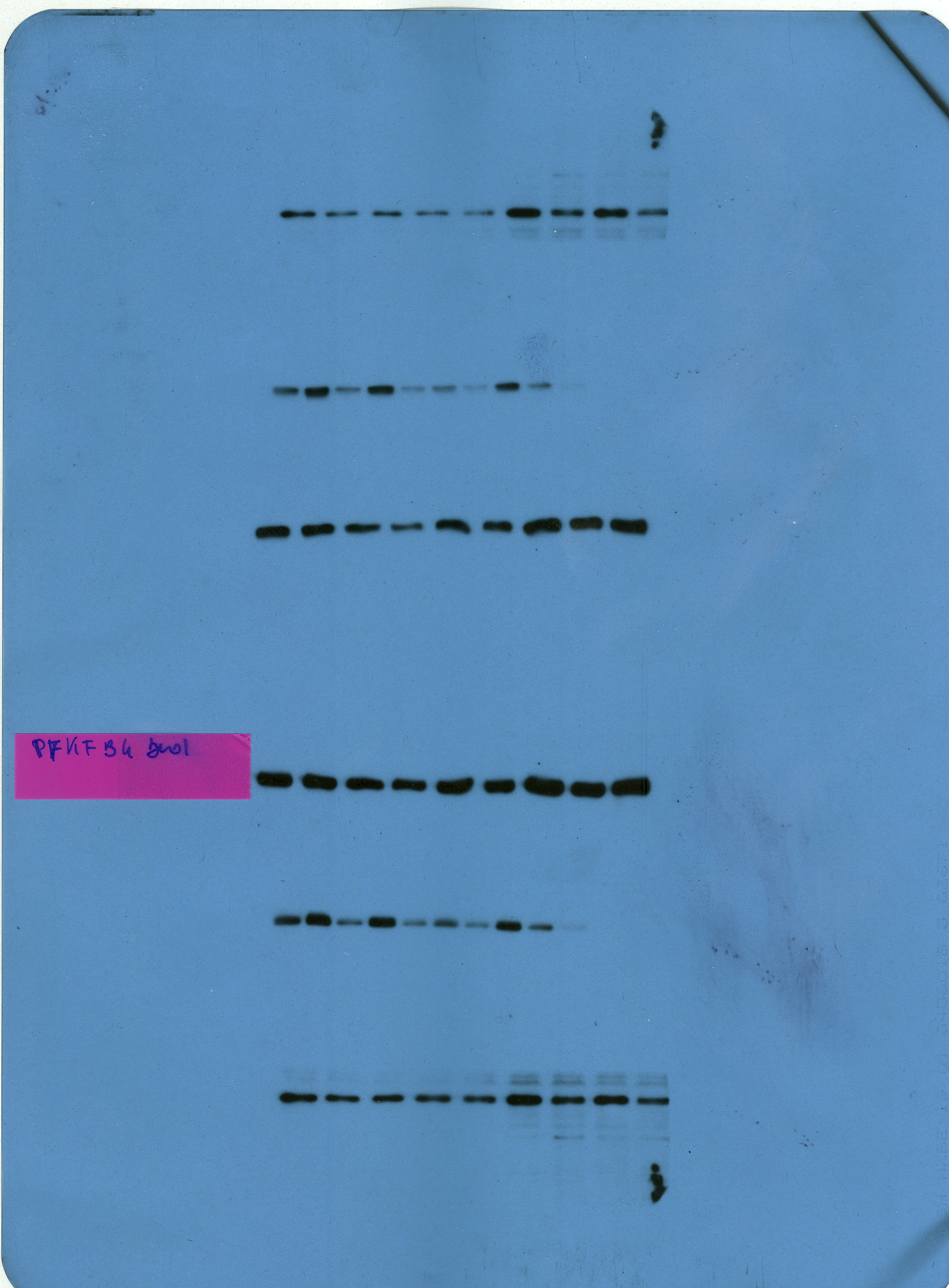

Supplement: Figure 4—source data 1. [file elife-86260-fig4-data1.zip › Fig 4A/Fig4A_pfkfb4 FILM/PFKFB4 entire0001.tif]

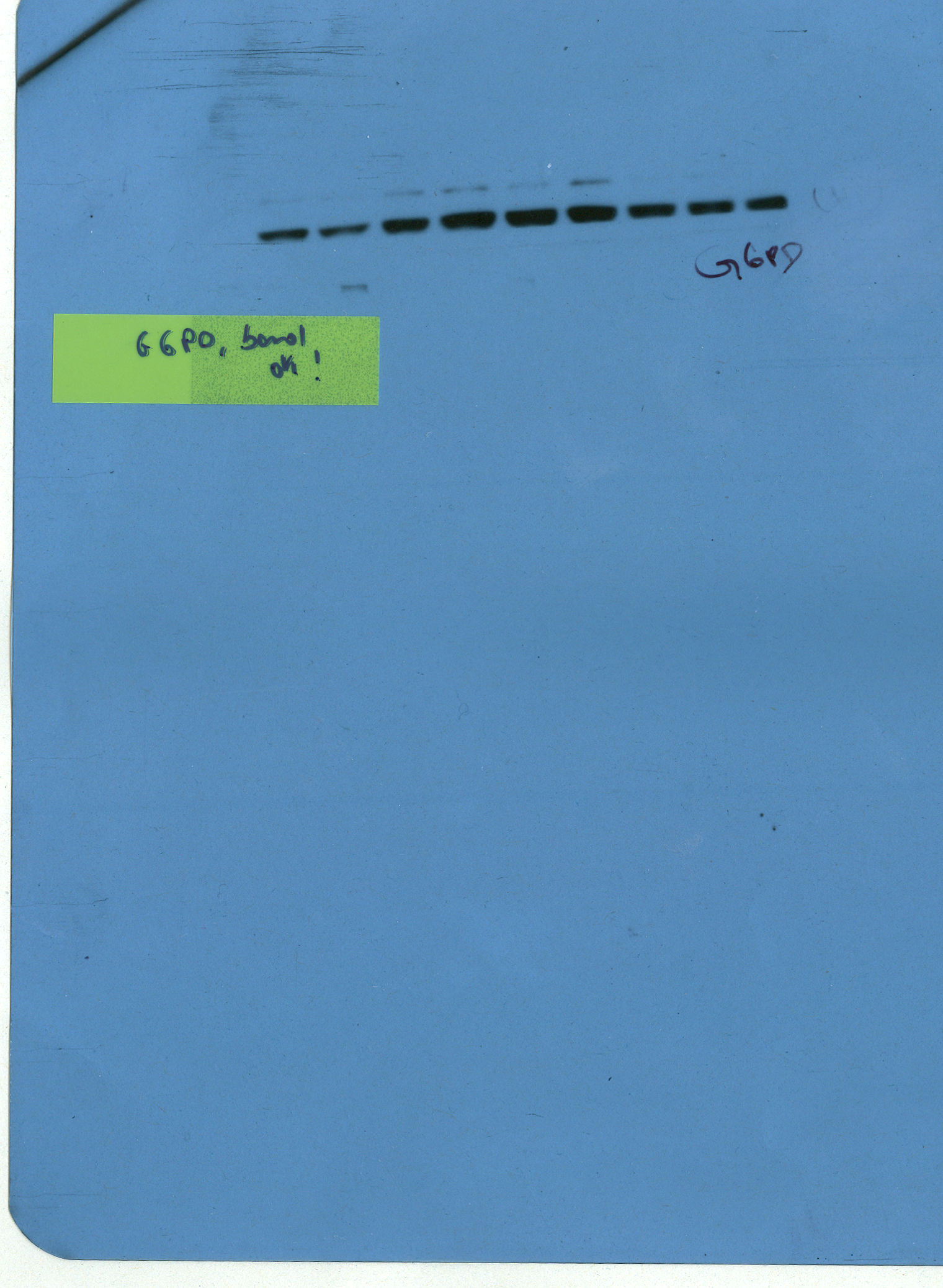

Supplement: Figure 4—source data 2. [file elife-86260-fig4-data2.zip › Fig 4C/g6pd CROP0001.tif]

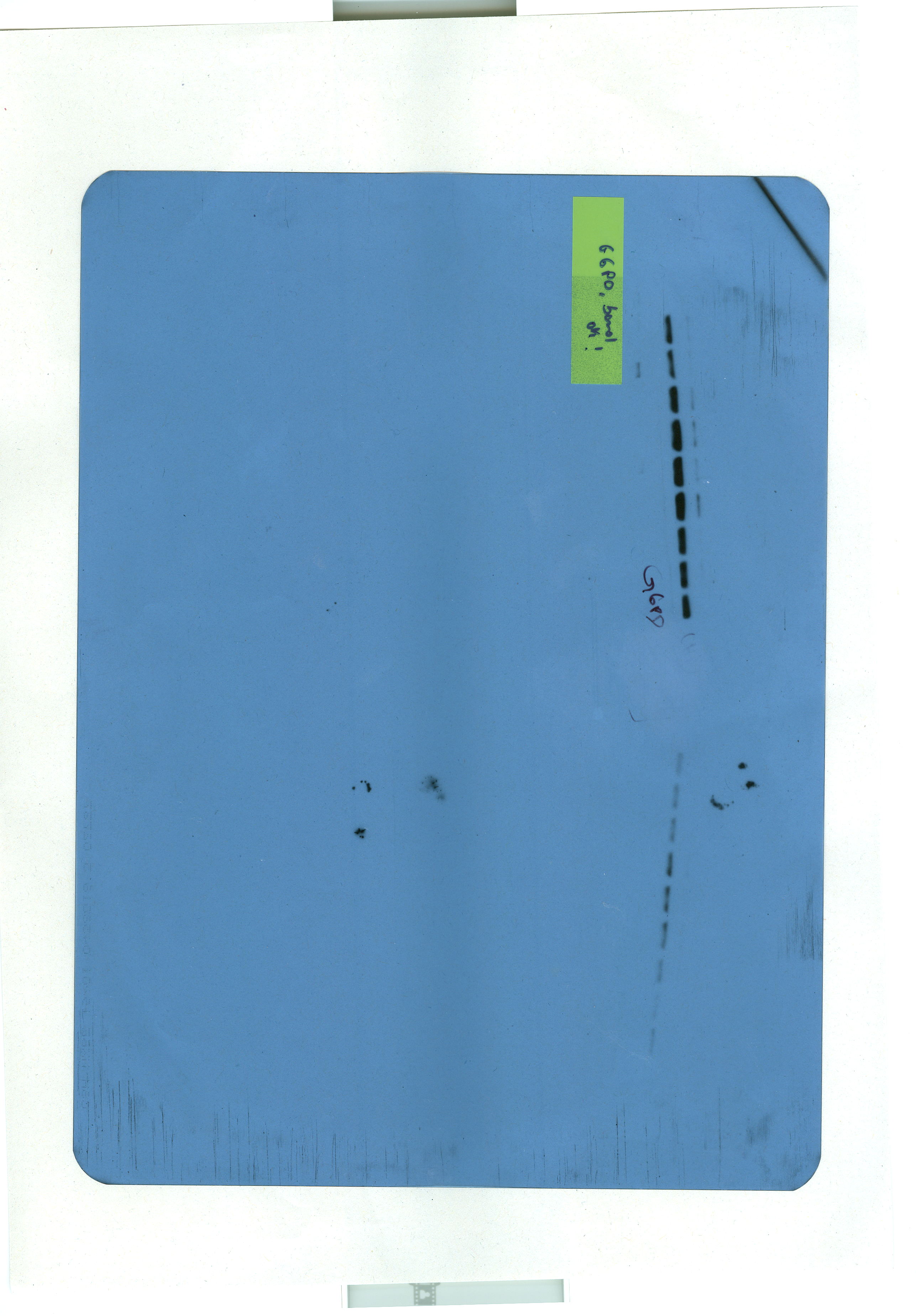

Supplement: Figure 4—source data 2. [file elife-86260-fig4-data2.zip › Fig 4C/G6PD new0002.tif]

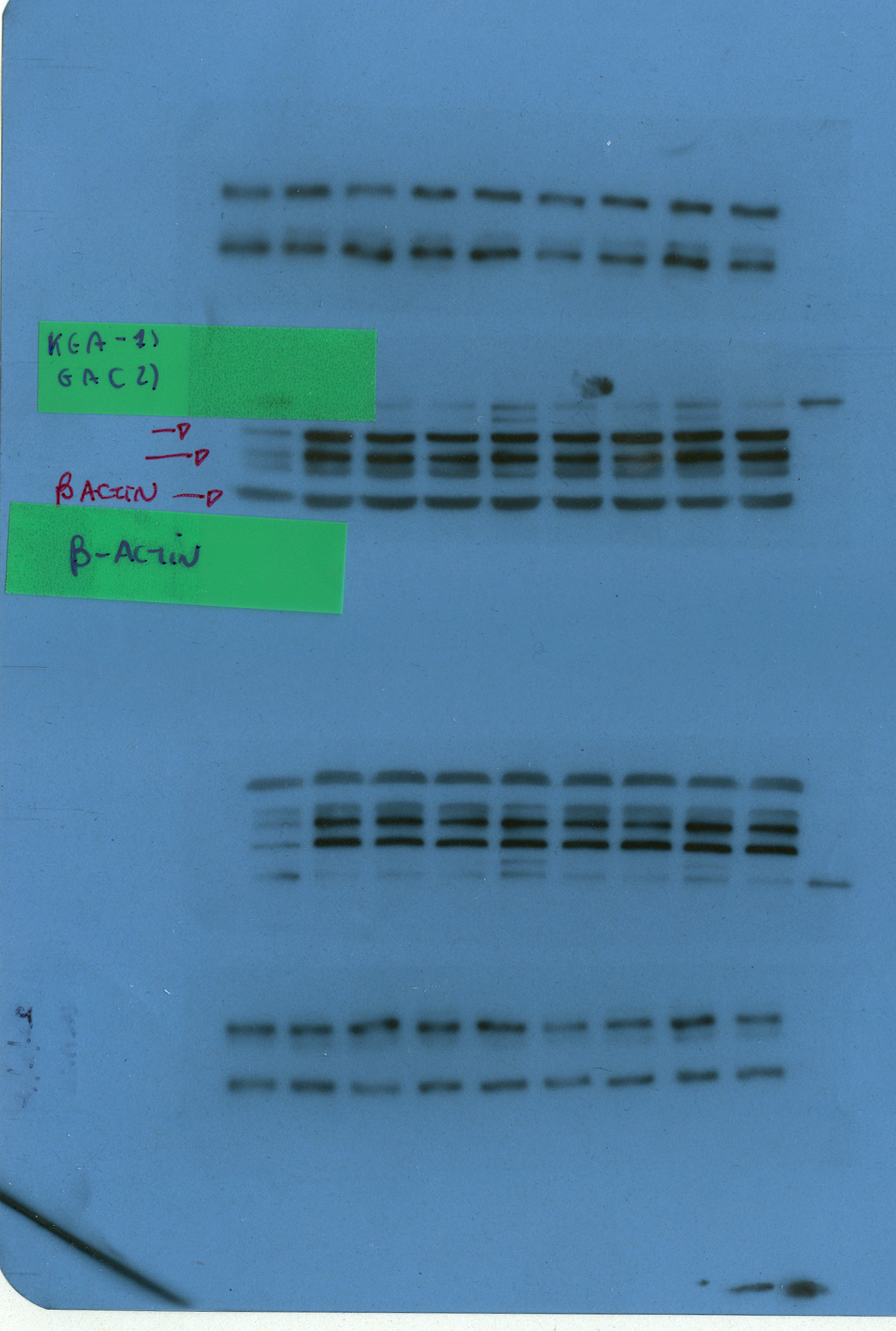

Supplement: Figure 5—source data 1. [file elife-86260-fig5-data1.zip › Fig 5B/KGA GAC ACTIN 0001.tif]
