# Supplementary material for: Autonomous metabolic reprogramming and oxidative stress characterize endothelial dysfunction in acute myocardial infarction
Source: eLife. 2023 Nov 28;12:e86260. doi: 10.7554/eLife.86260 (PMC10871716; doi:10.7554/eLife.86260)
Supplement: Reporting standard 1. [file elife-86260-repstand1.pdf]

Zodda et al.
Reporting Checklist
Biospecimen reporting for improved study quality (BRISQ) (Moore HM et al., DOI: 10.1002/cncy.20147)

Biospecimen type

Blood vessels

Anatomical site

Coronary arteries

Disease status of patients

Cardiovascular disease

Clinical characteristics of patients

Acute myocardial infarctionl patients

Vital State of patients

Alive

Clinical diagnosis of patients

Acute myocardial infarction

Pathology diagnosis

Acute myocardial infarction

Collection mechanism

Coronary artery catheterization

Type of stabilization

Heparin

Type of long-term preservation

Exvivo culture

Constitution of preservative

Complete endothelial culture medium (cell culture) and CECM+10% dimethyl sulfoxide (cryopreservation at -80 ºC)

Storage temperature –

37 ºC (cell culture), -80 ºC or liquid nitrogen (cryopreserved samples).

Storage duration

In culture: 72 h. Cryopreserved (liquid nitrogen): indefinite.

Shipping temperature

-170 ºC

Composition assessment & selection

100% endothelial cells
